# Interplay Between the American Diabetes Association’s ABC Targets for Diabetes, Insulin Resistance Indices, and Dyslipidemia in Indian Type 2 Diabetes Patients

**DOI:** 10.7759/cureus.60268

**Published:** 2024-05-14

**Authors:** Pramod Tripathi, Diptika Tiwari, Thejas Kathrikolly, Anagha Vyawahare, Baby Sharma, Malhar Ganla, Maheshkumar K, Venugopal Vijayakumar, Banshi Saboo, Nidhi S Kadam

**Affiliations:** 1 Department of Research, Freedom from Diabetes Research Foundation, Pune, IND; 2 Department of Management and Exercise Science, Freedom from Diabetes Clinic, Pune, IND; 3 Department of Physiology, Government Yoga and Naturopathy Medical College & Hospital, Chennai, IND; 4 Department of Yoga, Government Yoga and Naturopathy Medical College & Hospital, Chennai, IND; 5 Department of Endocrinology, Diabetes Care and Hormone Clinic, Ahmedabad, IND

**Keywords:** diabetes type 2, dyslipidemia, abc targets for diabetes, diabetes recommendations, beta cell function, insulin resistance

## Abstract

Background

The increasing incidence of type 2 diabetes (T2D) in India underscores the pressing need for effective management strategies. Meeting the American Diabetes Association (ADA) ABC targets for diabetes (glycated hemoglobin (HbA1c), blood pressure, and serum low-density lipoprotein cholesterol (LDL-C)) is crucial for effectively managing T2D, as it reflects the optimal control of key metabolic parameters. Insulin resistance (IR) and impaired beta cell function (BCF) have been found to have a significant impact on glycemic control, lipid metabolism, and hypertension, contributing to the complex cardiovascular risk profile of patients with T2D. This study aimed to explore the association between ABC targets for diabetes, IR, BCF, and dyslipidemia in a cross-sectional cohort of T2D patients.

Methods

This retrospective study examined data from 681 T2D patients with comorbid hypertension and dyslipidemia. The patients were part of a one-year online lifestyle intervention program for diabetes management at the Freedom from Diabetes Clinic in Pune, India, between January 2021 and December 2022. Baseline data (at the time of enrollment in the program) on medical history and anthropometric and biochemical parameters were retrospectively extracted from medical records and used to assess ABC targets and other clinical parameters. The ABC targets for diabetes include three goals: an HbA1c level of less than 7.0%, a blood pressure level of less than 140/90 mmHg, and an LDL-C level of less than 100 mg/dL. Homeostatic Model Assessment of Insulin Resistance (HOMA-IR), Homeostatic Model Assessment of Beta Cell Function (HOMA-B), and Quantitative Insulin Sensitivity Check Index (QUICKI) were calculated using standard formulas.

Results

Cross-sectional analysis at baseline showed that 152 (22.3%) participants met all three ABC targets, 306 (45.0%) and 183 (26.8%) participants met two or one targets, respectively, and 40 (5.9%) did not meet any of the ABC targets. Participants meeting all three targets showed significantly lower IR, higher sensitivity (HOMA-IR, median 2.1; QUICKI, median 0.34), higher BCF (HOMA-B, median 62.9), and healthier lipid profiles (mg/dL) (total cholesterol, median 126; triglycerides, median 114; and non-high-density lipoprotein (HDL), median 84) than those who did not meet any of the ABC targets (HOMA-IR, median 3.4; QUICKI, median 0.31; HOMA-B, median 31.7; total cholesterol, median 221; triglycerides, median 187; and non-HDL, median 182) (p < 0.01). A significant association was observed between lower BMI (< 25 kg/m^2^), lower IR (HOMA-IR <2.5), and meeting all three ABC targets (p < 0.01). No significant association was observed between the duration of diabetes and ABC target status (p > 0.1). Lower IR was identified as a predictor of achievement of all three ABC targets (p < 0.01).

Conclusion

This study highlights the significance of meeting ABC targets for diabetes in relation to not only a better lipid profile but also lower IR and higher BCF. These preliminary findings provide novel insights into the interplay between IR, BCF, dyslipidemia, and meeting ABC targets in an Indian T2D population. These findings highlight the need for effective diabetes management strategies and improved patient outcomes, considering factors such as BMI and IR indices.

## Introduction

Type 2 diabetes (T2D) is a chronic disease that is projected to affect over 700 million individuals worldwide by 2045, primarily owing to the rise in obesity and unhealthy lifestyles [1,2]. According to the American Diabetes Association (ADA), an effective approach for managing T2D includes maintaining optimal glycemic control and achieving the recommended ABC targets of glycated hemoglobin (HbA1c) <7%, blood pressure <140/90 mmHg, and low-density lipoprotein cholesterol (LDL-C) <100 mg/dL [3-5]. Available evidence demonstrates that achieving these targets can significantly decrease the risk of microvascular and macrovascular complications [6]. Despite recommendations from numerous clinical trials and guidelines, the management of T2D and its associated comorbidities, such as dyslipidemia and hypertension, in real-world settings remains suboptimal [7,8].

Insulin resistance (IR) and beta cell dysfunction are essential for T2D development and increase the risk of cardiovascular complications by affecting lipid metabolism [9,10]. They can also interfere with blood pressure regulation, potentially contributing to hypertension via mechanisms such as increased angiotensin II and aldosterone activity and oxidative stress [11,12]. The interconnected relationship between IR and beta cell function (BCF) highlights their multifaceted impact on glycemic control, dyslipidemia, and hypertension in individuals with T2D [10,11]. Optimal glycemic control, blood pressure, and lipid profiles are crucial for reducing the risk of cardiovascular disease. However, the available literature on the factors contributing to the non-attainment of these targets is limited [13].

India, which is home to the world’s second-largest population of people living with diabetes, faces considerable obstacles, as demonstrated by the fact that only a quarter of individuals have reached the recommended glycemic targets, and the percentage of patients meeting the recommended blood pressure targets is even worse [7,8]. However, there is a lack of evidence on the extent to which these ABC targets are met and the factors that contribute to them in the unique context of India’s demographics. The current study aimed to understand the baseline status of ABC targets and their association with IR, BCF, and dyslipidemia in an Indian population with T2D.

## Materials and methods

Study design and setting

This cross-sectional study was conducted using retrospective records from the electronic database of the Freedom from Diabetes Clinic in Pune, India. This study included 681 patients with T2D who were enrolled in a diabetes management program at the clinic between January 2021 and December 2022. Baseline data from the participants representing a single time point was used in the study.

Ethical consideration

This study was approved by the Institutional Ethics Committee (approval number FFDRF/IEC/2024/7). Owing to the retrospective design of the study, the requirement for informed consent from the participants was waived by the ethics committee.

Study criteria

Eligibility criteria included participants aged ≥18 years with a confirmed diagnosis of T2D without any significant diabetes-related complications, such as heart disease, diabetic nephropathy, or chronic illnesses, including liver and/or renal diseases. In light of the study’s objective, we included participants who had dyslipidemia and hypertension as comorbid conditions and were simultaneously taking medications for their management. However, participants taking insulin were excluded from the study. This was due to the calculation of the Homeostatic Model Assessment of Insulin Resistance (HOMA-IR), Homeostatic Model Assessment of Beta Cell Function (HOMA-B), and Quantitative Insulin Sensitivity Check Index (QUICKI), which utilized fasting insulin as one of the parameters for the calculations.

As all eligible participants were included in the analysis, sampling was not necessary.

Data collection and assessment of anthropometric and biochemical parameters

The de-identified baseline data of all eligible participants (N = 681) were retrospectively extracted from the database management system of the clinic. The collected data included anthropometry (height and weight), medical history (date of diabetes diagnosis, comorbidities, and medication status), and biochemical parameters (HbA1c, fasting blood sugar levels (FBSL), postprandial blood sugar, fasting insulin, and lipid profile). HOMA-IR and QUICKI were used to quantify the degree of IR and beta-cell secretory capacity. HOMA-B is an index of insulin secretory function derived from fasting plasma glucose and insulin concentrations. The HOMA-IR, HOMA-B, and QUICKI indices were calculated using standard formulas [14,15]. To assess ABC target status, appropriate cutoff values were applied to the baseline data for HbA1c (<7.0%), blood pressure (<140/90 mmHg), and LDL-C (<100 mg/dL). Based on these criteria, the participants were divided into four groups: Group A met all three targets, Group B met any two targets, Group C met any one target, and Group D did not meet any of the targets.

Statistical analysis

All statistical analyses were performed using IBM SPSS Statistics for Windows, Version 21.0 (Released 2012; IBM Corp., Armonk, NY, USA). Continuous variables are presented as means and standard deviations (for variables that follow a normal distribution) or medians and IQRs (for variables with a skewed distribution). Categorical variables are reported as frequencies and percentages. For continuous variables exhibiting skewed distributions, nonparametric tests, specifically the Kruskal-Wallis test and Mann-Whitney U test, were used to test the differences between more than two groups and between two groups, respectively. The association between categorical variables was tested using the chi-square test. Binary logistic regression was conducted to investigate the factors that independently predicted meeting all three ABC targets. The level of statistical significance was set at p < 0.05.

## Results

Sociodemographic characteristics of the study population

Table 1 describes the sociodemographic parameters of the population. Of the 681 participants, the proportion of male and female participants was 446 (65.5%) and 235 (34.5%), respectively (Table 1). The mean age of participants was 57 ± 9.1 years. The median duration of diabetes, weight, BMI, HbA1c level, FBSL, and fasting insulin level were 11.1 years (IQR, 6.8-16.8 years), 65 kg (IQR, 65.0-84.0 kg), 26.8 kg/m^2^ (IQR, 24.3-29.6 kg/m^2^), 7.4% (IQR, 6.8-8.5%), 126 mg/dL (IQR, 107-154 mg/dL), and 8.7 µU/mL (IQR, 5.7-13.4 µU/mL), respectively. Most participants were married (633; 93%) and had a family history of diabetes (511; 75%). Sixty-eight percent of participants had obesity (BMI ≥25 kg/m^2^). Additionally, the median medication duration for diabetes, dyslipidemia, and hypertension was 11.1 years (IQR, 6.4-16.9 years), 4.4 years (IQR, 2.0-9.3 years), and 10.4 years (IQR, 5.5-16.4 years), respectively. IR (HOMA-IR ≥2.5) and poor insulin sensitivity (QUICKI <0.34) were observed in 382 (56.1%) and 441 (64.8%) patients, respectively.

**Table 1:** Distribution of sociodemographic parameters in the study population

Variable	Parameter	Frequency (%)
Age group	Less than 50 years	159 (23.3)
	More than 50 years	522 (76.7)
Gender	Male	446 (65.5)
	Female	235 (34.5)
Marital status	Married	633 (93.0)
	Unmarried	17 (2.5)
	Divorced or separated	5 (0.7)
	Widowed	26 (3.8)
Education status	Graduation and below	373 (54.8)
	Post-graduation and above	308 (45.2)
Occupation	Salaried	400 (58.7)
	Retired	140 (20.6)
	Homemaker	107 (15.7)
	Others (students, preferred not to disclose)	34 (5.0)
Family history of diabetes	Both maternal and paternal	126 (18.5)
	Only maternal	172 (25.3)
	Only paternal	181 (26.6)
	Sibling	35 (5.1)
	None	167 (24.5)
Diabetes duration	Less than 10 years	285 (41.9)
	More than 10 years	396 (58.1)

ABC target status

**Figure 1:**
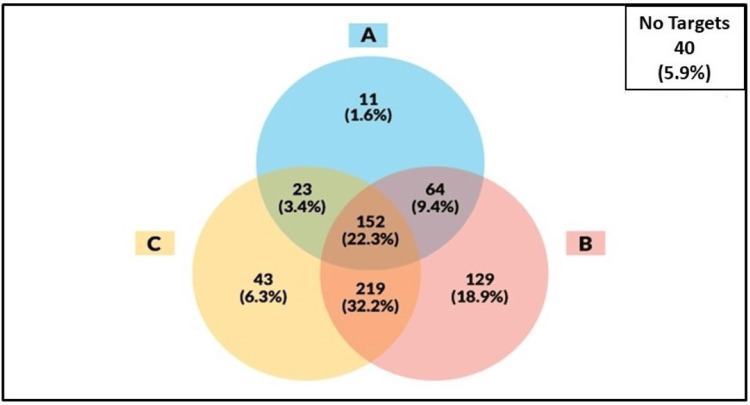
Overlap of ABC target status (one target, two targets, three targets, and no target) in patients with T2D with a focus on (A) HbA1c, (B) blood pressure, and (C) LDL-C HbA1c, glycated hemoglobin; LDL-C, low-density lipoprotein cholesterol; T2D, type 2 diabetes

Of the 681 T2D patients, 152 (22.3%) met all three ABC targets (Group A), whereas 306 (45%) and 183 (26.8%) met two (Group B) and one of the targets (Group C), respectively, despite being on medication for diabetes, hypertension, and dyslipidemia. Notably, 40 patients (5.9%) did not meet any of the ABC targets (Group D). Further analysis revealed a significant overlap between the groups of participants who met two targets and those who met only one of the targets (Figure 1). When examining participants who met dual targets, we observed that meeting targets for blood pressure and LDL-C led to higher rates of success (219 (32.2%)) than meeting HbA1c-Blood pressure (64 (9.4%)) and HbA1c-LDL-C targets (23 (3.4%)). Among the participants who met only one target, meeting the blood pressure targets demonstrated the highest success rate, followed by meeting the LDL-C and HbA1c targets (Figure 1). The pooled rates for meeting the HbA1c, blood pressure, and LDL-C targets were 250 (36.7%), 564 (82.8%), and 427 (64.2%), respectively.

Variation in IR, insulin sensitivity, and BCF based on ABC targets

**Figure 2:**
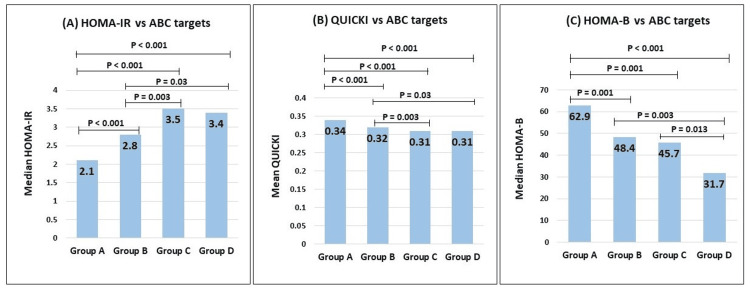
Variation in IR and sensitivity parameters of (A) HOMA-IR, (B) QUICKI, and (C) HOMA-B based on ABC targets status Group A: three targets (HbA1c, blood pressure, and LDL-C); Group B: two targets (HbA1c and blood pressure/HbA1c and LDL-C/blood pressure and LDL-C); Group C: one target (HbA1c/blood pressure/LDL-C); Group D: no target HbA1c, glycated hemoglobin; HOMA-B, Homeostatic Model Assessment of Beta Cell Function; HOMA-IR, Homeostatic Model Assessment of Insulin resistance; IR, insulin resistance; LDL-C, low-density lipoprotein cholesterol; QUICKI, Quantitative Insulin Sensitivity Check Index

We performed an analysis comparing the groups for IR indices based on ABC target status (Figure 2). The results indicated significantly lower IR (HOMA-IR) in Group A compared to Groups B, C, and D (p < 0.001). Additionally, participants in Group A showed higher sensitivity (QUICKI) and better BCF (HOMA-B) than Groups B, C, and D (p < 0.01). Furthermore, participants from Group B showed lower IR and better sensitivity than Groups C and D and better BCF than Group D (p < 0.05). Group C participants showed better BCF than Group D (p < 0.05).

Variation in BMI and lipid profile based on ABC targets 

The differences in BMI among the participants based on their ABC target profile showed a significantly lower BMI in Group A compared to Groups B, C, and D (p < 0.01). Additionally, the differences in lipid profiles among participants were examined, revealing a markedly healthier lipid profile at baseline in Group A than for Groups B, C, and D (p < 0.01). Furthermore, participants from Group B demonstrated significantly better lipid profiles than those in Groups C and D (p < 0.01). Similarly, participants in Group C displayed a significantly healthier lipid profile than those in Group D (p < 0.01) (Table 2).

**Table 2:** Association between BMI, lipid profile, and ABC target status Data for BMI and lipid profile are presented as median (IQR). The BMI and lipid profiles within and between the groups were compared using the Kruskal-Wallis test and the Mann-Whitney U test. ^a^ Significantly different than Group B at p-value <0.01 ^b^ Significantly different than Group C at p-value <0.01 ^c^ Significantly different than Group D at p-value <0.01 Group A: three targets (HbA1c, blood pressure, and LDL-C); Group B: two targets (HbA1c and blood pressure/HbA1c and LDL-C/blood pressure and LDL-C); Group C: one target (HbA1c/blood pressure/LDL-C); Group D: no target HbA1c, glycated hemoglobin; LDL-C, low-density lipoprotein cholesterol; non-HDL, non-high-density lipoprotein

Parameters	BMI and lipid profile based on ABC target status			
	Group A	Group B	Group C	Group D
Total N = 681	N = 152	N = 306	N = 183	N = 40
BMI (kg/m^2^)	25.9 (23.4-28.6)^a, b, c^	26.8 (24.1-29.4)	26.9 (25.0-30.4)	28.4 (25.3-30.7)
Total cholesterol (mg/dL)	126 (108-145)^a, b, c^	139 (114-165)^b, c^	192 (161-227)^c^	221 (190-249)
Triglyceride (mg/dL)	114 (80-154)^a, b, c^	124 (97-168)^b, c^	164 (116-218)^c^	187 (143-247)
Non-HDL (mg/dL)	84 (65-100)^a, b, c^	95 (76-127)^b, c^	150 (125-179)^c^	182 (146-206)

Factors associated with ABC targets

Table 3 presents the odd ratios and predictors for achieving all three ABC targets. Significant parameters from bivariate associations were entered into the regression model. Odds ratios from the chi-square analysis indicated that a lower BMI (<25 kg/m^2^), lower IR (HOMA-IR <2.5), and higher insulin sensitivity (QUICKI ≥0.34) were significantly associated with achieving all three ABC targets. Age, gender, education status, occupation, family history of diabetes, and diabetes duration were not significantly associated with the ABC target status (p > 0.1). The adjusted odds ratios showed that only lower IR predicted the achievement of all three ABC targets (p < 0.01).

**Table 3:** Predictors of meeting ABC targets at baseline HOMA-IR, Homeostatic Model Assessment of Insulin Resistance; QUICKI, Quantitative Insulin Sensitivity Check Index

Variables	Odds ratio (95% CI)	p-Value	Adjusted odds ratio (95% CI)	p-Value
BMI				
Obese (≥25 kg/m^2^) (ref)				
Non-obese (<25 kg/m^2^)	1.47 (1.17-1.8)	0.001	1.45 (0.98-2.15)	0.059
HOMA-IR				
≥2.5 (ref)				
<2.5	1.64 (1.39-1.9)	<0.001	2.74 (1.48-5.07)	0.001
QUICKI				
<0.34 (ref)				
≥0.34	1.61 (1.31-1.97)	<0.001	0.88 (0.47-1.62)	0.683

## Discussion

The present study aimed to investigate the cross-sectional prevalence and factors associated with meeting ABC targets among participants with T2D at baseline and to evaluate variations in IR, sensitivity, and BCF among those meeting different numbers of targets. Interestingly, our results showed that only 152 (22.3%) T2D participants met all three ABC targets, whereas 306 (45%) and 183 (26.8%) met two or one of these targets, respectively. These findings differ from those of previous studies that reported varying prevalence rates of ABC target achievement among T2D patients, with some studies demonstrating lower rates than ours, as discussed below.

The considerable disparity in the attainment of the targets was higher than the results of the Indian Council of Medical Research-India Diabetes study, which revealed a lower proportion of T2D patients (7.7%) successfully reaching all three of the ABC targets. However, this study was a population-based survey encompassing 113,043 adults with self-reported diabetes living in both rural and urban settings, and not all had comorbid hypertension and dyslipidemia [7]. In contrast, another study involving South Asian individuals, utilizing two successive cross-sectional population-based surveys in 2010-2011 (n = 16,288) and 2015-16 (n = 14,587), reported that 24% of the patients initially achieved all three targets, with this percentage subsequently increasing to 28% [16]. These conclusions align with the findings of the current study, which showed consistent trends in the accomplishment of diabetes care targets in South Asian populations. Additionally, our report also highlights that, despite being on medication for all three comorbid conditions, only one-fifth of the population met all three targets.

Studies on the achievement of ABC targets in T2D patients have been conducted worldwide. In a study conducted in Iran, the proportion of patients with T2D who achieved all three ABC targets increased from 23.2% in 2014 to 42.1% in 2017 [17]. Additionally, a retrospective study of 1,900 patients with T2D in two five-year periods (2010-2014 and 2015-2019) showed a significant increase in the attainment of all three ABC targets in the latter period [18]. However, these findings were not consistent with reports from other countries, such as Kuwait, where only 7.4% of patients achieved all three ABC targets [19]. Furthermore, a study of 465 Brazilian adults with diabetes by Dos Reis et al. [20] found that only 12.5% of the patients achieved ABC targets, which was lower than the findings of our study. Again, this could be due to the fact that these studies included diabetes patients irrespective of their medication status for comorbid hypertension and dyslipidemia.

According to a meta-analysis of 24 studies involving 369,251 individuals from 20 countries, the achievement rates of the recommended targets in T2D, as outlined by the ADA, the European Association for the Study of Diabetes, and the National Institute for Health and Care Excellence, were suboptimal. The pooled rates for glycemic control, blood pressure, and LDL-C were 42.8%, 29.0%, and 49.2%, respectively [21], which differed from our findings of 36.7%, 82.8%, and 64.2% for HbA1c, blood pressure, and LDL-C levels, respectively. Higher HbA1c control was noted in Europe and North America, while the latter also showed higher blood pressure target attainment, which was similar to our study. Furthermore, our findings on meeting two and one of the targets were consistent with those of a study conducted by Anjana et al. [16], which showed similar results of 42% and 28%, respectively, but contrasted with those of Dos Reis et al. [20], who reported a lower rate of 32.2% and a higher rate of 35.5% for attaining two and one ABC target, respectively.

Upon analyzing the factors associated with the attainment of targets, we observed that a higher BMI negatively impacted the attainment of ABC targets, which aligns with the findings of Larry et al. [17], Dos Reis et al. [20], and So et al. [22]. However, our study did not reveal a significant relationship between factors such as age, female gender, higher educational status, shorter diabetes duration, and the likelihood of achieving ABC targets, as reported by Anjana et al. [16], Larry et al. [17], Dos Reis et al. [20], and So et al. [22].

Previous studies have linked IR and sensitivity (measured by HOMA-IR, QUICKI, HOMA-B, and oral glucose tolerance test) to various health markers such as glycemic control, blood pressure, and dyslipidemia [10,11,23,24]. However, the collective impact of these factors on achieving ABC targets remains unclear, which is essential for maintaining glycemic control, reducing cardiovascular risk, and preventing complications in T2D patients. Our study found that those who met all three ABC targets at baseline had better IR, sensitivity, and BCF than those who met dual, single, or no targets. To our knowledge, this study is the first to establish a relationship between ABC targets and IR, insulin sensitivity, and BCF in the Indian population.

We further studied the relationship between ABC targets and dyslipidemia management. Our findings revealed that, as the number of ABC targets increased, there was a corresponding decrease in total cholesterol, triglycerides, and non-high-density lipoprotein cholesterol in T2D patients, which is consistent with the findings of Kitaoka et al. [13], who also reported that a higher number of ABC targets was associated with healthier lipid profiles in T2D patients. This emphasizes the potential benefits of achieving ABC targets to improve lipid profiles and overall cardiovascular health.

Our findings underscore the need for comprehensive treatment approaches for T2D patients. These approaches should concentrate on helping patients attain all three ABC targets and emphasize their importance in diabetes management. Despite this, our study has certain limitations, including self-reported blood pressure and a single-center design, which may restrict the applicability of our conclusions. Additionally, the lack of details on medication dosing schedule prevented us from exploring their implications for meeting the ABC targets. Moreover, information on patient compliance with their medication regimen was not accessible, and because of the study’s retrospective design, it could not be collected. Future prospective studies are needed to shed light on these associations.

## Conclusions

Our study highlights the importance of meeting the ABC targets of the ADA for effectively managing T2D in the Indian population. Participants who met all three targets demonstrated significantly lower IR, higher BCF, and healthier lipid profiles than those who did not meet any targets. These findings underscore the significance of comprehensive metabolic control in reducing cardiovascular risk in patients with T2D. This further emphasizes the need for effective diabetes management strategies and improved patient outcomes, considering factors such as BMI and IR indices. Future research should explore the impact of personalized treatment plans and lifestyle interventions on ABC target achievement in T2D patients.
